# X Chromosome Evolution in Cetartiodactyla

**DOI:** 10.3390/genes8090216

**Published:** 2017-08-31

**Authors:** Anastasia A. Proskuryakova, Anastasia I. Kulemzina, Polina L. Perelman, Alexey I. Makunin, Denis M. Larkin, Marta Farré, Anna V. Kukekova, Jennifer Lynn Johnson, Natalya A. Lemskaya, Violetta R. Beklemisheva, Melody E. Roelke-Parker, June Bellizzi, Oliver A. Ryder, Stephen J. O’Brien, Alexander S. Graphodatsky

**Affiliations:** 1 Institute of Molecular and Cellular Biology, SB RAS, Lavrentiev Ave. 8/2, Novosibirsk 630090, Russia; zakal@mcb.nsc.ru (A.I.K.); polina.perelman@gmail.com (P.L.P.); alex.makunin@gmail.com (A.I.M.); lemnat@mcb.nsc.ru (N.A.L.); bekl@mcb.nsc.ru (V.R.B.); graf@mcb.nsc.ru (A.S.G.); 2 Synthetic Biology Unit, Novosibirsk State University, Pirogova Str. 1, Novosibirsk 630090, Russia; 3 The Royal Veterinary College, University of London, Royal College Street, London NW1 0TU, UK; dmlarkin@gmail.com (D.M.L.); mfarrebelmonte@gmail.com (M.F.); 4 Animal Sciences Department, College of ACES, University of Illinois at Urbana-Champaign, IL 61801, USA; avk@illinois.edu (A.V.K.); jjohnso@illinois.edu (J.L.J.); 5 Frederick National Laboratory of Cancer Research, Leidos Biomedical Research, Inc., Frederick, MD 21702, USA; melodyr@mail.nih.gov; 6 Catoctin Zoo and Wildlife Preserve, Thurmont, MD 21788, USA; rapunta@yahoo.com; 7 San Diego Zoo Institute for Conservation Research, 15600 San Pasqual Valley Road, Escondido, CA 92027, USA; oryder@sandiegozoo.org; 8 Theodosius Dobzhansky Center for Genome Bioinformatics, Saint-Petersburg State University, Sredniy Av. 41A, Saint-Petersburg 199034, Russia; lgdchief@gmail.com; 9 Oceanographic Center, Nova Southeastern University, Fort Lauderdale 3301 College Ave, Fort Lauderdale, FL 33314, USA

**Keywords:** Pecora, Ruminantia, cattle bacterial artificial chromosome (BAC) clones, fluorescent in situ hybridization (FISH), intrachromosomal rearrangements, centromere reposition, inversion

## Abstract

The phenomenon of a remarkable conservation of the X chromosome in eutherian mammals has been first described by Susumu Ohno in 1964. A notable exception is the cetartiodactyl X chromosome, which varies widely in morphology and G-banding pattern between species. It is hypothesized that this sex chromosome has undergone multiple rearrangements that changed the centromere position and the order of syntenic segments over the last 80 million years of Cetartiodactyla speciation. To investigate its evolution we have selected 26 evolutionarily conserved bacterial artificial chromosome (BAC) clones from the cattle CHORI-240 library evenly distributed along the cattle X chromosome. High-resolution BAC maps of the X chromosome on a representative range of cetartiodactyl species from different branches: pig (Suidae), alpaca (Camelidae), gray whale (Cetacea), hippopotamus (Hippopotamidae), Java mouse-deer (Tragulidae), pronghorn (Antilocapridae), Siberian musk deer (Moschidae), and giraffe (Giraffidae) were obtained by fluorescent in situ hybridization. To trace the X chromosome evolution during fast radiation in specious families, we performed mapping in several cervids (moose, Siberian roe deer, fallow deer, and Pere David’s deer) and bovid (muskox, goat, sheep, sable antelope, and cattle) species. We have identified three major conserved synteny blocks and rearrangements in different cetartiodactyl lineages and found that the recently described phenomenon of the evolutionary new centromere emergence has taken place in the X chromosome evolution of Cetartiodactyla at least five times. We propose the structure of the putative ancestral cetartiodactyl X chromosome by reconstructing the order of syntenic segments and centromere position for key groups.

## 1. Introduction

Despite the great variation in diploid number and high level of autosome reshuffling, the X chromosome of eutherian mammals is evolutionary conserved. The size and morphology of the X chromosome as defined by the position of the centromere is similar in most mammalian orders. Hypothetically, this unique conservation was guided by the establishment of a mechanism for dosage compensation in the therian ancestor [1]. The emergence of this mechanism is thought to have imposed evolutionary constraints on chromosomal rearrangements in the sex chromosome [1].

Classical cytogenetic techniques were used to describe morphology, centromere position, banding pattern, and heterochromatin distribution in a wide range of species. Comparative analysis has identified similar X chromosome morphology and G-banding patterns across species from different taxa (primates, pigs, camels, carnivores, perissodactyls) [2]. Comparative mapping of the X chromosome with gene-specific probes confirmed similarity in the gene order on the X chromosome of distantly related species (human, pig, horse, dog, cat) [3]. These studies provided strong evidence for Ohno’s rule, confirming genomic conservancy of eutherian X chromosomes. However, some notable exceptions in conservation phenomenon of X chromosome have been identified in Cetartiodactyla and Rodentia. The modified X chromosome structure in these orders is caused by inversions, changes in centromere position, heterochromatin expansion and autosome to sex chromosome translocations [4].

The order Cetartiodactyla exhibits great diversity of chromosome X morphology both within and between families. Note that in most eutherian orders only autosomal syntenic segments undergo reshuffling as shown by cross-species chromosome painting [5]. The exact mechanisms behind dynamic changes on X chromosome in Cetartiodactyla are unknown. Comparative chromosome painting with whole chromosome painting probes, including X, has been employed in several studies [6,7,8,9,10,11]. These studies showed that cetartiodactyl autosomes evolved through fission, fusion, and inversions. However, unlike autosomes, the sex chromosomes evolved through more complex chromosomal rearrangements involving reshuffling of conserved segments inside the chromosome, changes in centromere positions, heterochromatic variation, and autosomal translocations [12,13]. It is likely that centromere repositioning (shift) or so-called evolutionary new centromere phenomenon, reflecting a change of centromere position on the chromosome without a change in the gene order, also occurred in cetartiodactyl X chromosome evolution. So far it was shown only in primates, rodents and perissodactyls [14,15,16,17].

The structure of cetartiodactyl X chromosomes has been closely studied mainly in domestic species from the family Bovidae [13,18,19,20,21,22], and in a few wild species from the families Giraffidae, Cervidae, Antilocapridae and Hippopotamidae [6,23,24,25]. In previous studies, microdissection probes or arm-specific paints and several bacterial artificial chromosome (BAC) clones were used to detect intrachromosomal rearrangements. A recent investigation showed centromere repositioning and inversions in cetartiodactyl X chromosomes [25]. Interspecific X chromosome variation in the Cetartiodactyla has been a source of some controversy in the past [12]. The analysis of X chromosome rearrangements can be a potential source of phylogenetic information [12], but the X chromosome evolution in Cetartiodactyla has not yet been studied in detail.

In the present study, we report the comparative map of cetartiodactyl X chromosomes obtained by cross-species hybridization with the set of cattle BAC clones, and provide new data about X chromosome evolution in 10 cetartiodactyl families. Our analysis allows reconstruction of the ancestral X chromosome for major nodes of the cetartiodactyl tree and traces the rearrangements of X chromosome that have occurred during evolution within this order.

## 2. Materials and Methods

### 2.1. Species

The list of studied species with scientific and common names, diploid chromosome number, and source of cell lines is presented in the Table 1. All cell lines belong to the cell cultures collection of general biological purpose (No. 0310-2016-0002) of Institute of Molecular and Cellular Biology Siberian Branch of the Russian Academy of Sciences.

### 2.2. Chromosome Preparation

Metaphase chromosomes were obtained from fibroblast cell lines. Briefly, cells were incubated at 37 °C and 5% CO_2_ in medium αMEM (Sigma Aldrich Co., St. Louis, MO, USA) supplemented with 15% fetal bovine serum, 5% AmnioMAX-II complete (Gibco^TM^) and antibiotics (ampicillin 100 µg/mL, penicillin 100 µg/mL, amphotericin B 2.5 µg/mL). Metaphases were obtained by adding colcemid (0.02 mg/mL) and ethidium bromide (1.5 mg/mL) to actively dividing culture for 3–4 h. Hypotonic treatment was performed with 3 mM KCl, 0.7 mM sodium citrate for 20 min at 37 °C and followed by fixation with 3:1 methanol/glacial acetic acid (Carnoy’s) fixative. Metaphase chromosome preparations were made from a suspension of fixed fibroblasts, as described previously [26]. G-banding on metaphase chromosomes prior to fluorescence in-situ hybridization (FISH) was performed using standard procedure [27].

### 2.3. BAC Clones

Using the cattle genome assembly version from October 2011 (Baylor Btau_4.6.1/bosTau7) in UCSC Genome Browser [28], X chromosome-located BAC clones were manually chosen from the CHORI-240 BAC library from the “BACPAC Resource Center” (BPRC, the Children’s Hospital Oakland Research Institute in Oakland, CA, USA). To download information in the Genome Browser about the localization of BACs of appropriate size (length of insertion varied from 50–300 kb), a custom track in Browser Extensible Data (BED) format was created [29]. BAC clones with appropriate insert sizes (50–300 kbp) and genetic content (unique genes, less repetitive elements) were selected. BAC sequence conservation was estimated from phyloP data [30] in the human genome (“Conservation” track in GRCh37/hg19 assembly). Genome coordinates were converted from cow to human using the Batch Coordinate Conversion (liftOver tool) in UCSC Genome Browser. Thus, 73 BAC clones evenly distributed on cattle X chromosome (2–5 Mbp gaps) were selected. For each of the manually selected 73 BACs, we defined various genomic features selected to increase the probability of a clone to hybridize with metaphase spreads of distant cetartiodactyl species. To do so, we calculated protein coding genes, cattle genes orthologous to human, GC content, and repetitive sequences in each of the selected BAC clones. By using multiple alignments, including all available cetartiodactyl genomes, we calculated the nucleotide conservation scores and conserved elements using phastCons [31]. Then, we compared the characteristics of four BACs that had previously worked on distant species with all the 73 BACs by using the classification tree from the CART algorithm [32]. A total of 51 BACs were selected to have a high probability of hybridization to distant species. These BACs contained less than 48% of repetitive sequence and more than 20% of conserved elements. A subset of 26 of these BAC clones that were evenly distributed along the cattle X chromosome with a median distance of 5 Mb were hybridized on all cetartiodactyl species studied here. Table 2 lists the CHORI-240 cattle X chromosome BAC clones used in this study. A single BAC clone (CH240-316D2) is the same as used by Fröhlich et al. [25].

BAC DNA was isolated using the Plasmid DNA Isolation Kit (BiosSilica, Novosibirsk, Russia) and amplified with GenomePlex Whole Genome Amplification kit (Sigma-Aldrich Co., St. Louis, MO, USA). Labeling of BAC DNA was performed using GenomePlex WGA Reamplification Kit (Sigma-Aldrich Co., St. Louis, MO, USA) by incorporating biotin-16-dUTP or digoxigenin-dUTP (Roche, Basel, Switzerland). The quality of produced BAC probes was controlled by FISH localization on cattle chromosomes.

### 2.4. Fluorescence In-Situ Hybridization (FISH)

Dual-color FISH experiments on G-banded metaphase chromosomes were performed as described by Yang and Graphodatsky [26]. Digoxigenin-labeled and biotin-labeled probes were detected with Cy^TM^3 anti-digoxin (Jackson ImmunoResearch Laboratories, Inc., West Grove, PA), fluorescein avidin DCS, biotinilated anti-avidin D (Vector Laboratories, Inc., Burlingame, CA, USA), respectively. Images were captured with a Baumer Optronics CCD Camera (Baumer Ltd., Southington, CT, USA) mounted on an Olympus BX53 microscope (Olympus, Shinjuku, Japan) and processed using VideoTesT 2.0 Image Analysis System (Zenit, St. Petersburg, Russia).

### 2.5. Bioinformatics Analysis

An analysis in UCSC Genome Browser was performed to establish the order of CHORI-240 BAC clones on X chromosomes of one cetartiodactyl species (sheep) and four species from out-group mammalian orders (Perissodactyla, Primates, Rodentia). BAC positions in these genomes were obtained using Batch Coordinate Conversion (liftOver) in the UCSC Genome Browser that converts genome coordinates between assemblies. The cattle genome assembly (Bos_taurus_UMD3.1.1/bosTau8) was used as a reference. Sequences coordinates of all BAC clones were calculated in human (GR ch38/hg 38), mouse (GRC m38/mm10), rat (RGSC 6.0/rn6, except 386M8, which is disrupted in this genome), horse (Broad/equCab2), and sheep (ISGC Oar_v3.1/oviAri3) genomes.

### 2.6. Ancestral Chromosome Deduction

The morphology and conservative block orientation of the ancestral X chromosome were deduced using maximum parsimony by comparing X chromosomes across the top branches of Cetartiodactyla and assuming the most common variant to be ancestral for the order. Once the provisional ancestral chromosome was identified, we detected whether the extant X chromosome and the suggested ancestral form differ by inversions (change of BAC order) or/and by centromere repositioning (change of centromere position without change in BAC order).

## 3. Result

### 3.1. BACs Localization

We investigated the X chromosome structure across major branches of Cetartiodactyla represented by 18 species from four non-ruminant (Suidae, Camelidae, Eschrichtiidae (Cetacea), Hippopotamidae) and six ruminant (Tragulidae, Antilocapridae, Giraffidae, Moschidae, Cervidae, and Bovidae) families (Table 1). The order of 26 labeled cattle BAC clones was established on the X chromosomes of each of 18 species by a series of pairwise FISH experiments (Table 2). In total, comparative analyses of BAC orders across 18 species revealed three major chromosomal conservative segments, which were numbered and designated with colors used throughout the paper: X Syntenic Block 1 (13 BACs, XSB1, pink); X Syntenic Block 2 (seven BACs, XSB2, yellow), and; X Syntenic Block 3 (six BACs, XSB3, blue).

### 3.2. Intrachromosome Rearrangements

Comparative analysis of the order of BAC on the X chromosomes of 18 species identified three key scenarios that likely took place in the course of the cetartiodactyl X chromosomes’ evolution.

**Figure 1:**
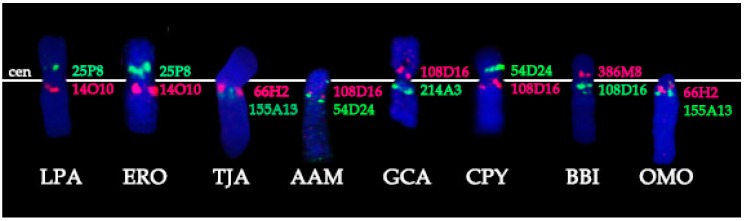
Centromere location (cen, white line) and positions of specific BAC clones (pink and green) on X chromosome of several cetartiodactyl species. Species three-letter codes are listed in Table 1.

**Figure 2:**
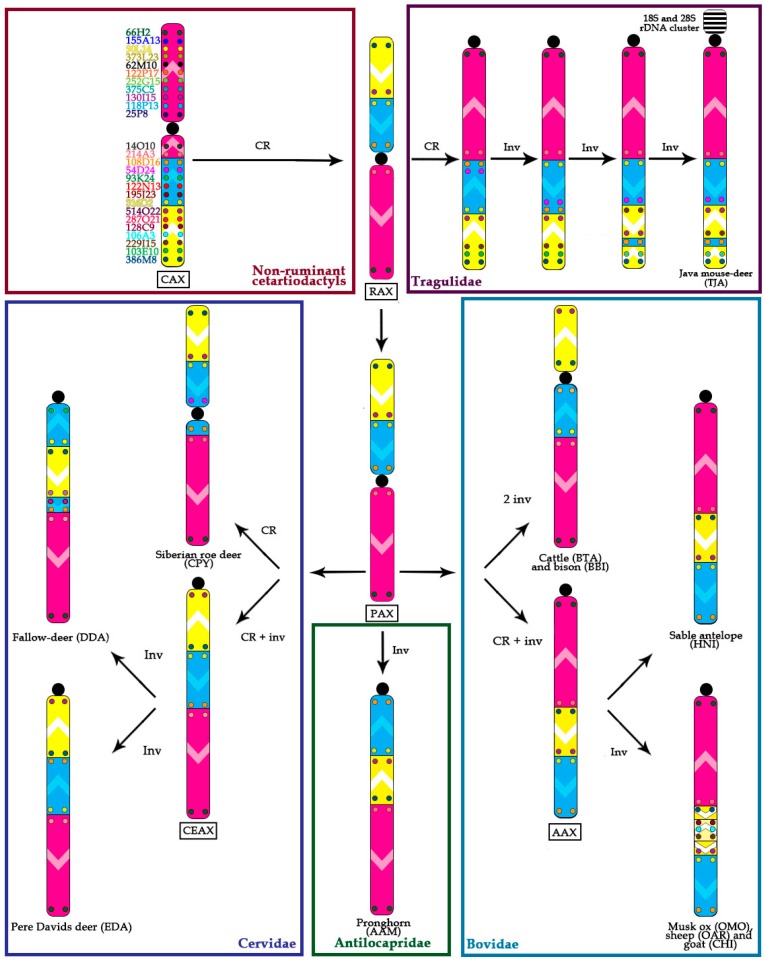
The scheme of evolutionary transformations of X chromosome in Cetartiodactyla. Chromosome rearrangements were identified by changes in BAC order. Three major conservative segments are designated by different colors: pink—X syntenic block 1; yellow—X syntenic block 2, and; blue—X syntenic block 3. Individual BAC clones are shown with a different color in small colored circles on corresponding conservative segment. Centromere position is indicated by a black circle. The orientation of the conservative segments is indicated by the white arrowhead. Ancestral associations are shown in black rectangle (Cetartiodactyla ancestral X (CAX), Ruminantia ancestral X (RAX), Pecora ancestral X (PAX), Antilopinae ancestral X (AAX), Cervinae ancestral X (CEAX)). CR: centromere reposition. Inv: inversion.

1. Conservation: no change in the BAC order and no change of the centromere position. We identified a group of four basal cetartiodactyl species (gray whale (ERO), common hippopotamus (HAM), alpaca (LPA), and pig (SSC)) that have an identical order of the BACs and the same relative position of the centromere (located in XSB1).
2. Centromere repositioning: conserved BAC order, changes in the centromere position. Centromere repositions have been shown in roe deer, and mouse-deer, resulting in metacentric (Siberian roe deer (CPY)) and acrocentric (Java mouse-deer (TJA)) X chromosomes, respectively. This event took place prior to a formation of some lineage specific ancestral chromosomes (RAX (Ruminant Ancestral X), AAX (Antilopinae Ancestral X), and CEAX (Cervinae Ancestral X)), indicating that centromere repositioning is one of the key rearrangements of the ruminant X: while maintaining a conserved order of the segments there was a displacement of the centromere (Figure 1).
3. Inversion: changes in the BAC order. Three kinds of inversions were identified: (A) syntenic block (SB) flip—this inversion reverses the orientation of the whole syntenic block (TJA, AAM, AAX, CEAX); (B) an inversion inside the syntenic block (goat (CHI), muskox (OMO)); (C) the exchange inversion—inversion that involves several BAC clones from two syntenic blocks (TJA, fallow deer (DDA)) (Figure 2).

Taken together, we found that inversions (paracentric and pericentric) and centromere shifts were key rearrangements in the course of X chromosome evolution in Cetartiodactyla. In addition to the described rearrangements, the nucleolar organizing region (containing clusters of 18S and 28S rDNA genes) were localized on the short arm of both X and Y sex chromosomes of the Java mouse-deer (TJA) [33].

### 3.3. Bioinformatic Analysis of Mammalian X Chromosomes

To evaluate the unique conservation of mammalian X chromosomes [3] we calculated the coordinates of 26 BAC clone sequences in four Boreoeutherian non-cetartiodactyl genomes represented by Euarchontoglires: human (Primates); mouse, and rat (Rodentia), and; Laurasiatheria: horse (Perissodactyla). We have observed that three X chromosome syntenic blocks (XSB) found in Cetartiodactyla are conserved in Laurasiatheria and also in Euarchontoglires, indicating common Boreoeutherian structure of the X chromosome. It was previously reported that human, horse, and pig X chromosomes have similar gene order [3]. In general, this observation was confirmed by liftOver analyses (Table 3). We have identified several small inversions in XSB1 (human, horse) and in XSB2 (horse) in comparison to CAX. Interestingly, XSB1 is the most derived segment outside of Cetartiodactyla, no rearrangements in BACs order in the cetartiodactyl species were detected within this block. According to our data, XSB2 is highly conserved in non-cetartiodactyl species, while in ruminants there are inversions inside of this syntenic block (CHI, OMO, sheep (OAR)) and exchange inversions between XSB2 and XSB3 (TJA and DDA).

We also aligned the BAC clone sequences to another cetartiodactyl genome, the domestic sheep. We observed the same BAC order as in all analyzed Caprini species except for a small inversion in XSB3. The FISH with relevant BAC clones confirmed the presence of this inversion in the sheep genome.

## 4. Discussion

### 4.1. Ancestral X Chromosome

The phenomenon of X chromosome conservation in eutherian mammals was first proposed by Susumu Ohno and was based solely on its size similarity across a wide range of species [1]. High similarity in G-banding pattern led to the hypothesis that not only size and gene content [34] but also gene order is conserved on the X chromosomes of most eutherian mammals, and this was later confirmed by fine gene mapping [3,35,36,37,38]. Remarkably, the submetacentric X chromosome morphology defined by the location of the centromere is also largely conserved across mammals. Some slight changes of otherwise conserved X chromosomes were observed in several orders, such as the difference in the distance between homologous genes between human and alpaca [39], or a shift in centromere position without a change of the gene order in Afrotheria [37]. Still, the lack or low level of rearrangements of the X chromosome in comparison to the active exchanges on autosomes during over 150 million years of eutherian evolution represents an interesting phenomenon. Comparative G-banding analysis had identified the classical chromosome X morphology and banding pattern common to most eutherian species [2]. Similar submetacentric morphology and gene order were also found in non-ruminant cetartiodactyls. A high level of X chromosome conservation was shown in Suinae [3,40], Tylopoda [41,42,43], and Cetacea [44]. Nevertheless, using G-banding analysis [4] and high-resolution mapping with BACs [25] or region specific probes, [12] intrachromosomal rearrangements were uncovered in Ruminantia species. Compared with the previous study, we have expanded the number of BACs to 26 and the species list to 18 in order to define conservative blocks and their orientation, to identify rearrangements across species, and to reconstruct the ancestral cetartiodactyl X chromosome. The analyses of BAC order across major families of Cetartiodactyla revealed three syntenic blocks on the X chromosome that in general correspond to the conserved segments reported by Fröhlich and coauthors [25].

**Figure 3:**
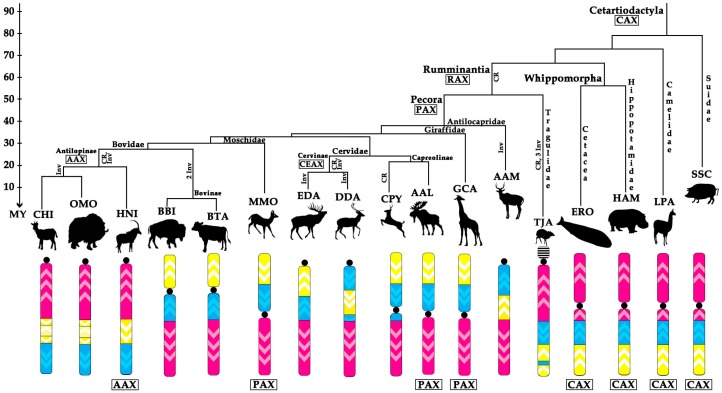
The structure of the Cetartiodactyla X chromosome depicted on the phylogenetic tree of the order (the tree topology from [47]) Major conservative segments are shown by yellow, blue, and pink. Centromere positions are designated by a black circle. White arrowheads show the orientation of the conservative segments. Ancestral associations are shown under X chromosomes (Cetartiodactyla ancestral X (CAX), Ruminantia ancestral X (RAX), Pecora ancestral X (PAX), Antilopinae ancestral X (AAX). MMO X chromosome is inverted here relatively to its cytogenetic orientation for presentation purposes [8].

Using available FISH and bioinformatic data on the order of cattle BACs in the genomes of different species, we were able to investigate the phenomenon of the conservation of the X chromosome in eutherian mammals represented by four superorders: Laurasiatheria; Euarchontoglires; Afrotheria, and; Xenarthra [45]. Three conserved syntenic blocks identified here can be traced in Boreoeutherians (Laurasiatheria and Euarchontoglires), and possibly in all eutherians, considering reports on Afrotheria X chromosome conserved gene order [37] (Table 2). The eutherian X chromosome ancestral condition (EUX) is represented by a submetacentric chromosome with the centromere located in XSB1. Bioinformatic analysis in outgroup species shows a common change of BAC order in XSB1 on human and horse X chromosomes. Supposedly, an inversion on EUX had occurred in the ancestor of Cetartiodactyla prior the radiation of this order. This ancestral condition was revealed in all non-ruminant cetartiodactyls and named here Cetartiodactyla Ancestral X (CAX). We confirmed the conservation of the X chromosome in basal branches of Cetartiodactyla. It occurs in Suidae (pig), Camelidae (alpaca), and Cetacea (gray whale) (Table 2 and Figure 3). Cetacea is a sister taxon to Hippopatamidae and is characterized by extremely conserved karyotypes across the whole infraorder and by uniform X chromosome morphology and banding pattern [11,46]. The Hippopotamidae X chromosome also displays the same morphology and the gene order [8,25]. However, it should be emphasized that there are some unresolved cases of the X chromosome changes in these basal groups that would require additional investigation, for example, the X chromosome of *Tayassu pecari* (Suinae, Taysuidae) has been changed due to a centromere reposition [40].

### 4.2. Ancestral Form of Ruminantia-Pecora X-Chromosome

Contrary to the conservation of the X chromosome in Suidae-Camelidae-Whippomorpha, we found that multiple rearrangements occurred during the radiation of other cetartiodactyl branches. We suggest that in the Ruminantia an ancestral centromere reposition led to changes of the X chromosome morphology from submetacentric to metacentric forming the Ruminantia Ancestral X-chromosome (RAX) (Figure 2 and Figure 3). Both ancestral forms (CAX and RAX) have same intrachromosomal structure and differ only by centromere position. The RAX form of the X chromosome is also preserved in many basal Pecora branches: Giraffidae (GCA); Moschidae (MMO); and in the Capreolini (AAL) subfamily of Cervidae. Only in the basal Pecoran family Antilocapridae, an inversion turned the ancestral metacentric X chromosome into an acrocentric element (Figure 2). Thus we expect that the Ancestral Ruminant and the Ancestral Pecoran X chromosomes have the same structure: RAX=PAX.

In the Tragulidae, the basal and the only non-Pecora ruminant group, we found a major centromere reposition resulted in the formation of an acrocentric X. Also, two kinds of inversions (SB-flip and synteny block exchange) affect syntenic block structure in the Tragulidae. These rearrangements create unique arrangement of the three syntenic blocks in the Java mouse-deer. This arrangement may occur across all tragulids, but requires confirmation in other *Tragulus* species.

### 4.3. Cervidae

There is a great variation in X chromosome morphology among cervids. Two cervid subfamilies, Capreolinae and Cervinae, exhibit a notably differing extent of sex chromosome conservation. The only detected rearrangement was a centromere shift in CPY. G-banding pattern comparison of Capreolinae X chromosomes otherwise indicates a uniform metacentric morphology [48] and suggests a similar disposition of conservative syntenic blocks. 

In contrast, Cervinae is characterized by a variety of rearrangements on the X chromosome: centromere repositioning, SB flips, and many inversions disrupting the XSB2. The Cervinae Ancestral X-chromosome (CEAX) was formed by a centromere reposition and a SB flip of XSB2. Inversions change this ancestral form in EDA by SB flip of XSB3 and in DDA by the splitting of XSB2 (Figure 2). Also in the same subfamily, a translocation of an autosome to the X chromosome was reported in several Muntiacini species [7,49,50,51,52]. In total, this indicates that the level of X chromosome variation is increased in Cervinae and is caused not only by inversions and centromere repositioning but also by autosome to sex chromosome translocations.

### 4.4. Bovidae

The family Bovidae includes two major branches: Bovinae and Antilopinae [53]. Earlier cytogenetic studies identified two types of morphological diversity of X chromosome in Bovidae: a caprine type (acrocentric, suni type) and a bovine type (submetacentric) [12,54]. The bovine type of X chromosome was likely formed from the ancestral pecoran X (PAX) by two inversions. This form is retained in cattle (BTA) and American bison (BBI). Cytogenetic data for other studied Bovinae species demonstrated same submetacentric X chromosome morphology [48]. There are independent autosome translocations in two branches (Tragelaphini and Bosephalini) altering the bovine type X chromosome [12,23,48,55,56]. The notable exceptions are the Bubalina lineage, oryx and kudu (Tragelaphilini), whose X chromosomes have acrocentric morphology (designated as eland-type acrocentric based on eland, kudu, and nyala X chromosomes [12]).

Centromere reposition and inversion events resulted in the formation of an acrocentric Antilopinae Ancestral X-chromosome (AAX) (Figure 2) from PAX. Therefore the X of the sable antelope (HNI) could likely represent an ancestral form for all Antilopinae. Moreover, comparative analyses based on published karyotypes supports the theory that the X chromosome in antelopes is largely conserved, retaining the same morphology and banding pattern [48,57]. The exceptions are autosome to X chromosome translocations found in several Antilopini species [48,58]. In the Caprini lineage there is an additional inversion within the XSB3 (OMO, CHI, OAR). The bioinformatic and FISH analyses of X chromosome of OAR indicated that the inversion between 128C9 and 229I15 is an apomorphic phylogenetic marker for Caprini.

### 4.5. X Chromosome Rearrangements

All X chromosome rearrangements discovered here are in agreement with the current phylogenetic tree (Figure 3), and some of them could be used as cytogenetic markers for different Cetartiodactyla groups. Therefore, we suggest that our BAC clone set can serve as a precise instrument for a further search for cytogenetic X chromosome markers in Bovidae. The independent autosome to sex chromosome translocations that occurred in several Bovidae and Cervidae branches require special attention because they increase the previously identified rapid rate of evolution of the structure of the cetartiodactyl X chromosome [7,12,49,50,51,52,55,56].

The BAC clones that mark the borders of three conserved segments delineate regions of frequent chromosome rearrangements in cetartiodactyl X, indicating a breakpoint reuse phenomenon [59]. Several BAC clones were involved at least twice in the intrachromosomal rearrangements found here, suggesting breakpoint reuse: 108D16 and 214A3; 514O22 and 316D2; 229I15 and 103E10. We found that the regions surroundings these BACs in the cattle genome are often gene sparse. It was previously shown that chromosomal regions with evolutionary breakpoint in amniotes are enriched for structural variations (segmental duplications, copy number variants, and indels), retrotransposons, zinc finger genes, and single nucleotide polymorphisms [60]. Further investigation is required to find precise points of evolutionary chromosome breakage on the Cetatiodactyla X and to define common genomic features underlying chromosome rearrangements.

Another mammalian order characterized by the increased rate of X chromosome evolution is Rodentia. Heterochromatin expansion, amplification of tandem repeats, inversions [61], centromere reposition [62], and autosome to sex chromosome translocations [63] were shown to be involved in rearrangements of X chromosome in rodents. Comparative chromosomal analysis of X chromosomes was performed by microdissection in the *Microtus* genus. Rubtsov with coauthors postulated that intrachromosomal rearrangements are associated with large clusters of intrachromosomal duplications and/or repeated DNA sequences which were present in ancestral species but have subsequently disappeared during evolution [61]. We hypothesize that similar processes were involved in evolution of X chromosome in Ruminantia. Some genomic events possibly took place in the ruminant ancestor that launched multiple chromosomal rearrangements of the conservative eutherian X chromosome. Insertions of mobile repetitive elements such as long and short interspersed nuclear elements (LINE and SINE were probably involved in synteny breaks on this sex chromosome [64]. It is possible that this transforming genomic event had happend in or around the XSB2 area which demonstrates highest rate of inversions in Ruminantia.

In total, nine paracentric, two pericentric inversions, and five centromere reposition events have been revealed in Cetartyodactyla X chromosome evolution based on the analysis of 18 species. The eutherian and cetartiodactyl ancestral X differ only by one small inversion; one additional rearrangement is proposed to derive the Ruminantia ancestral X (RAX). Most other identified rearrangements happend during the remaining 55 million years of ruminant’s radiation. The cow X chromosome was formed by at least two rearrangements that distinguish it from PAX, corresponding to a rate of rearrangements of approximately 1 per 15 million years. This is comparable to 1 rearrangement per 10 million years postulated for autosomal evolution among most mammalian orders found by chromosome painting [65]. These findings are consistent with the rate of X chromosome evolution in Ruminantia being at least twice as high as in X chromosomes of average eutherian mammalian group.

## 5. Conclusions

High-resolution X chromosome maps of cetartiodactyl species provide unique information about evolution of intrachromosomal rearrangements. Three conserved syntenic blocks have been identified. We postulate that inversions and centromere repositioning were two key types of rearrangements in course of cetartiodactyl X chromosome evolution. The detailed analysis of the BAC order across multiple species by FISH mapping and bioinformatic analysis allowed the reconstruction of a putative cetartiodactyl ancestral X chromosome. The basal cetartiodactyl group of non-ruminants (pigs, camels, whales, and hippos) share this metacentric ancestral type of X chromosome. The submetacentric ancestral Ruminantia X chromosome was likely formed by simple centromere shift but it retained the ancestral intrachromosomal structure. Currently observed X chromosome morphological variation was formed by inversions and centromere repositioning during 55 million years of ruminant evolution. Chromosome rearrangements supporting the taxonomic status of ruminant families and subfamilies were found by mapping 26 BAC clones specific to the X chromosome. The rate of X-specific rearrangements in Ruminantia significantly exceeds that among eutherian mammals.

## Figures and Tables

**Table 1:** List of cetartiodactyl species included in this study and their characteristics.

Scientific Name, Abbreviation	Code	Common Name	Family	Diploid Number	Source of Cell Line
*Sus scrofa*	SSC	Pig	Suidae	38, XX	IMCB SB RAS, Novosibirsk-1*
*Lama pacos*	LPA	Alpaca	Camelidae	74, XY	2*
*Eschrihtius robustus*	ERO	Gray whale	Eschrichtiidae (Cetacea)	44, XY	[11]
*Hippopotamus amphibius*	HAM	Common hippopotamus	Hippopotamidae	36, XY	[8]
*Tragulus javanicus*	TJA	Java mouse-deer	Tragulidae	32, XY	Frozen Zoo (San Diego Zoo’s Conservation Research, San Diego, CA, USA)
*Antilocapra americana*	AAM	Pronghorn	Antilocapridae	58, XY	[10]
*Giraffa camelopardalis*	GCA	Giraffe	Giraffidae	30, XY	[8]
*Moschus moschiferus*	MMO	Siberian musk deer	Moschidae	58, XY	[8]
*Dama dama*	DDA	Fallow deer	Cervidae, Cervinae	68, XX	Catoctin Wildlife Preserve and Zoo, Maryland, USA
*Elaphurus davidianus*	EDA	Pere David’s deer		68, XX	3*
*Alces alces*	AAL	Eurasian elk	Cervidae, Capreolinae	68, XX	IMCB SB RAS, Novosibirsk
*Capreolus pygargus*	CPY	Siberian roe deer		70, XX	IMCB SB RAS, Novosibirsk
*Ovibos moschatus*	OMO	Muskox	Bovidae, Antilopinae	48, XX	IMCB SB RAS, Novosibirsk
*Capra hircus*	CHI	Goat		60, XX	Catoctin Wildlife Preserve and Zoo, Maryland, USA
*Ovis aries musimon*	OAR	Sheep		54, XX	Catoctin Wildlife Preserve and Zoo, Maryland, USA
*Hippotragus niger*	HNI	Sable antelope		60, XX	3*
*Bison bison*	BBI	American bison	Bovidae, Bovinae	60, XX	4*
*Bos taurus*	BTA	Cattle		60, XX	IMCB SB RAS, Novosibirsk

1*: IMCB SB RAS - Institute of Molecular and Cellular Biology Siberian Branch of the Russian Academy of Sciences. 2*: The cell line is established by William Nash (Laboratory of Genomic Diversity, NCI, Frederick, MD, USA). The sample provided by Camelid Research Group (Oregon State University, Corvallis, OR, USA). 3*: Sample provided by Mitchell Bush (Conservation and Research Center, National Zoological Park, Front Royal, VA, USA). Cell line is established in the Laboratory of Genomic Diversity (NCI, Frederick, MD, USA). 4*: The sample is provided by Douglas Armstrong (Henry Doorly Zoo, Omaha, NE, USA). Cell line is established in the Laboratory of Genomic Diversity (NCI, Frederick, MD, USA).

**Table 2:** CHORI-240 BAC’s order on cetartiodactyl X chromosomes. The color of the cells corresponds to a certain conservative syntenic segment.

No.	BAC’s Order and Localization on Cattle X Chromosome			CHORI (CH-240) BACs Localization on Cetartiodactyl X Chromosomes														
				Domestic Pig, SSC	Alpaca, LPA	Gray Whale, ERO	Common Hippopota-mus, HAM	Java Mouse-Deer, TJA	Pronghorn, AAM	Giraffe, GCA	Siberian Roe Deer, CPY	Eurasian Elk, AAL	Fallow Deer, DDA	Pere David’s Deer, EDA	Muskox, OMO	Goat, CHI	Sheep, OAR	Sable Antelope, HNI
1	X syntenic block 2 (XSB2)	CH240-514O22	Start 1949353, End 2129088	66H2	66H2	66H2	66H2	66H2	108D16	386M8	386M8	386M8	93K24	514O22	66H2	66H2	66H2	66H2
2		CH240-287O21	Start 7324034, End 7488466	155A13	155A13	155A13	155A13	155A13	54D24	103E10	103E10	103E10	122N13	287O21	155A13	155A13	155A13	155A13
3		CH240-128C9	Start 8233624, End 8391009	90L14	90L14	90L14	90L14	90L14	93K24	229I15	229I15	229I15	195J23	128C9	90L14	90L14	90L14	90L14
4		CH240-106A3	Start 13345128, End 13540519	373L23	373L23	373L23	373L23	373L23	122N13	106A3	106A3	106A3	316D2	106A3	373L23	373L23	373L23	373L23
5		CH240-229I15	Start 13805346, End 13950311	62M10	62M10	62M10	62M10	62M10	195J23	128C9	128C9	128C9	386M8	229I15	62M10	62M10	62M10	62M10
6		CH240-103E10	Start 20150516, End 20286173	122P17	122P17	122P17	122P17	122P17	316D2	287O21	287O21	287O21	103E10	103E10	122P17	122P17	122P17	122P17
7		CH240-386M8	Start 33395588, End 33587168	252G15	252G15	252G15	252G15	252G15	514O22	514O22	514O22	514O22	229I15	386M8	252G15	252G15	252G15	252G15
8	X syntenic block 3 (XSB3)	CH240-108D16	Start 48672324, End 48917704	375C5	375C5	375C5	375C5	375C5	287O21	316D2	316D2	316D2	106A3	108D16	375C5	375C5	375C5	375C5
9		CH240-54D24	Start 53219586, End 53351583	130I15	130I15	130I15	130I15	130I15	128C9	195J23	195J23	195J23	229I15	54D24	130I15	130I15	130I15	130I15
10		CH240-93K24	Start 57734547, End 57947720	118P13	118P13	118P13	118P13	118P13	106A3	122N13	122N13	122N13	287O21	93K24	118P13	118P13	118P13	118P13
11		CH240-122N13	Start 62228039, End 62371946	25P8	25P8	25P8	25P8	25P8	229I15	93K24	93K24	93K24	514O22	122N13	25P8	25P8	25P8	25P8
12		CH240-195J23	Start 62982639, End 63183460	14O10	14O10	14O10	14O10	14O10	103E10	54D24	54D24	54D24	54D24	195J23	14O10	14O10	14O10	14O10
13		CH240-316D2	Start 68490278, End 68678635	214A3	214A3	214A3	214A3	214A3	386M8	108D16	108D16	108D16	108D16	316D2	214A3	214A3	214A3	214A3
14	X syntenic block 1 (XSB1)	CH240-214A3	Start 84397606, End 84521707	108D16	108D16	108D16	108D16	316D2	214A2	214A3	214A3	214A3	214A3	214A3	386M8	386M8	386M8	386M8
15		CH240-14O10	Start 85224265, End 85389684	54D24	54D24?	54D24	54D24	195J23	14O9	14O10	14O10	14O10	14O10	14O10	103E10	103E10	103E10	103E10
16		CH240-25P8	Start 90681870, End 90861947	93K24	93K24	93K24	93K24	122N13	25P7	25P8	25P8	25P8	25P8	25P8	128C9	128C9	128C9	229I15
17		CH240-118P13	Start 92264186, End 92429310	122N13	122N13	122N13	122N13	93K24	118P12	118P13	118P13	118P13	118P13	118P13	106A3	106A3	106A3	106A3
18		CH240-130I15	Start 95938488, End 96135558	195J23	195J23	195J23	195J23	54D24	130I14	130I15	130I15	130I15	130I15	130I15	229I15	229I15	229I15	128C9
19		CH240-375C5	Start 103959199, End 104119579	316D2	316D2	316D2	316D2	514O22	375C4	375C5	375C5	375C5	375C5	375C5	287O21	287O21	287O21	287O21
20		CH240-252G15	Start 108195394, End 108349350	514O22	514O22	514O22	514O22	287O21	252G14	252G15	252G15	252G15	252G15	252G15	514O22	514O22	514O22	514O22
21		CH240-122P17	Start 110284444, End 110450903	287O21	287O21	287O21	287O21	128C9	122P16	122P17	122P17	122P17	122P17	122P17	316D2	316D2	316D2	316D2
22		CH240-62M10	Start 111125731, End 111275450	128C9?	128C9	128C9	128C9?	106A3	62M9	62M10	62M10	62M10	62M10	62M10	195J23	195J23	195J23	195J23
23		CH240-373L23	Start 117191008, End 117371368	106A3	106A3	106A3	106A3	229I15	373L22	373L23	373L23	373L23	373L23	373L23	122N13	122N13	122N13	122N13
24		CH240-90L14	Start 126821940, End 127050706	229I15	229I15	229I15	229I15	108D16	90L13	90L14	90L14	90L14	90L14	90L14	93K24	93K24	93K24	93K24
25		CH240-155A13	Start 128339848, End 128504608	103E10	103E10	103E10	103E9	103E10	155A12	155A13	155A13	155A13	155A13	155A13	54D24	54D24	54D24	54D24
26		CH240-66H2	Start 141101222, End 141358968	386M8	386M8	386M8	386M7	386M8	66H1	66H2	66H2	66H2	66H2	66H2	108D16	108D16	108D16	108D16

**Table 3:** CHORI-240 (CH-240) BAC’s order on mammalian chromosomes X. Conservative syntenic segments are colored in pink, yellow and blue.

No.	Laurasiatheria						Euarchontoglires					
	BAC Clones in Cattle Genome		BAC Clones in Sheep Genome		BAC Clones in Horse Genome)		BAC Clones in Human Genome		BAC Clones in Mouse Genome		BAC Clones in Rat Genome	
1	514O22	Start 1949353	66H2	Start 10045822	66H2	Start 8367618	66H2	Start 12497685	118P13	Start 7554450	25P8	Start 1711095
		End 2129088		End 10306770		End 8624882		End 12794877		End 7697987		End 1907049
2	287O21	Start 7324034	155A13	Start 19299853	155A13	Start 16543677	155A13	Start 22069138	62M10	Start 9209615	375C5	Start 4672236
		End 7488466		End 19464920		End 16698622		End 22228453		End 9317028		End 4863802
3	128C9	Start 8233624	373L23	Start 28630482	373L23	Start 24698243	373L23	Start 31328065	122P17	Start 10195810	252G15	Start 10936630
		End 8391009		End 28810179		End 24857300		End 31509266		End 10370080		End 11107682
4	106A3	Start 13345128	62M10	Start 34891275	62M10	Start 30220096	62M10	Start 31328065	252G15	Start 12644301	122P17	Start 13483272
		End 13540519		End 35037294		End 30342071		End 31509266		End 12803364		End 14335671
5	229I15	Start 13805346	122P17	Start 35738657	122P17	Start 30907267	122P17	Start 38298814	375C5	Start 18235010	62M10	Start 14415064
		End 13950311		End 35910824		End 31039631		End 38458494		End 18480200		End 14541523
6	103E10	Start 20150516	252G15	Start 37830134	252G15	Start 32879937	252G15	Start 40611820	25P8	Start 20507324	118P13	Start 15650399
		End 20286173		End 37981845		End 33007527		End 40767797		End 20696050		End 15784402
7	386M8	Start 33395588	375C5	Start 41973255	375C5	Start 36512266	375C5	Start 45036869	514O22	Start 23213727	130I15	Start 22235385
		End 33587168		End 42128838		End 36698919		End 45234319		End 23316229		End 22435973
8	108D16	Start 48672324	130I15	Start 49649383	25P8	Start 38190847	25P8	Start 47047149	287O21	Start 41535889	66H2	Start 27957571
		End 48917704		End 49847996		End 38327897		End 47226311		End 41677049		End 28439737
9	54D24	Start 53219586	118P13	Start 52564228	118P13	Start 39580949	118P13	Srart 49122932	128C9	Start 42491010	155A13	Start 40510641
		End 53351583		End 52727917		End 39734268		End 49608099		End 42653374		End 40710667
10	93K24	Start 57734547	25P8	Start 54170178	130I15	Start 44962739	130I15	Start 53053920	106A3	Start 47802786	373L23	Start 53052665
		End 57947720		End 54331345		End 45135718		End 53291737		End 48012093		End 53277814
11	122N13	Start 62228039	14O10	Start 59810734	14O10	Start 52316685	14O10	Start 70333575	229I15	Start 48279488	14O10	Start 70503930
		End 62371946		End 59977176		End 52471298		End 70530493		End 48451406		End 70671925
12	195J23	Start 62982639	214A3	Start 60702841	214A3	Start 53269385	214A3	Start 71438703	103E10	Start 57106307	214A3	Start 71468323
		End 63183460		End 60821565		End 53389760		End 71567090		End 57244888		End 71575467
13	316D2	Start 68490278	386M8	Start 80094458	108D16	Start 76549898	108D16	Start 97540872	386M8	Start 71145260	108D16	Start 100451494
		End 68678635		End 80283568		End 76701833		End 97704621		End 71388925		End 100747362
14	214A3	Start 84397606	103E10	Start 93391997	93K24	Start 81725518	54D24	Start 103662230	373L23	Start 84771898	93K24	Start 107378470
		End 84521707		End 93531761		End 81893517		End 103838933		End 84970050		End 107552526
15	14O10	Start 85224265	287O21	Start 101734836	54D24	Start 83362409	93K24	Start 105651553	14O10	Start 100669857	54D24	Start 109470944
		End 85389684		End 101892691		End 83480210		End 105782858		End 100840304		End 109865654
16	25P8	Start 90681870	128C9	Start 102635193	122N13	Start 86318687	122N13	Start 109356460	214A3	Start 101583273	122N13	Start 113344277
		End 90861947		End 102791210		End 86434114		End 109486477		End 101676469		End 113475228
17	118P13	Start 92264186	106A3	Start 107701336	195J23	Start 86961327	195J23	Start 110108649	108D16	Start 130409135	195J23	Start 114041201
		End 92429310		End 107885819		End 87148727		End 110310972		End 130602938		End 114226114
18	130I15	Start 95938488	229I15	Start 108152302	316D2	Start 89122764	316D2	Start 112670755	93K24	Start 136717423	316D2	Start 116629155
		End 96135558		End 108300381		End 89285710		End 112844328		End 136874331		End 116812891
19	375C5	Start 103959199	514O22	Start 111218064	514O22	Start 93641183	514O22	Start 117884744	54D24	Start 138738349	514O22	Start 121570459
		End 104119579		End 111402064		End 93800197		End 118065524		End 138862889		End 121690157
20	252G15	Start 108195394	316D2	Start 115697759	287O21	Start 97957842	287O21	Start 123321223	122N13	Start 142065201	287O21	Start 127687918
		End 108349350		End 115869258		End 98099669		End 123489384		End 142200659		End 127828202
21	122P17	Start 110284444	195J23	Start 118153613	128C9	Start 98755966	128C9	Start 124334066	195J23	Start 142770604	128C9	Start 128883616
		End 110450903		End 118356580		End 98902048		End 124491573		End 142959710		End 129042712
22	62M10	Start 111125731	122N13	Start 118952818	106A3	Start 102828316	106A3	Start 129437403	316D2	Start 145340226	106A3	Start 134638127
		End 111275450		End 119094106		End 103004015		End 129628777		End 145507925		End 134841751
23	373L23	Start 117191008	93K24	Start 122240643	229I15	Start 103242991	229I15	Start 129926486	130I15	Start 152167108	229I15	Start 135116351
		End 117371368		End 122443675		End 103384683		End 130107731		End 152363913		End 135281818
24	155A13	Start 128339848	54D24	Start 124299513	103E10	Start 108619397	103E10	Start 136556505	155A13	Start 157177353	103E10	Start 159580103
		End 128504608		End 124431515		End 108750001		End 136681177		End 157384607		End 159734497
25	66H2	Start 141101222	108D16	Start 129843594	386M8	Start 119476270	386M8	Start 150502313	66H2	Start 167378561		
		End 141358968		End 130091258		End 119683931		End 150728735		End 167730488		
